# Varicella in San Francisco

**Published:** 1871-10

**Authors:** 


Varicella in San Francisco.—Chicken-pox has been prevailing lately in San Francisco in an aggravated form, some cases so near to small-pox in character as to have been reported as such to the Board of Health.

---

Among the notable deaths which have taken place within the last few months are those of Dr. Liegeois, Vice-Professor of the Paris Faculty, Dr. Thomas Hawkes Tanner, of England, the well known author, Dr. George C. Blackman, Professor of Surgery in the Medical College of Ohio, also well known as an eminent teacher and writer.

